# Upregulation of *I*_h_ expressed in IB4-negative Aδ nociceptive DRG neurons contributes to mechanical hypersensitivity associated with cervical radiculopathic pain

**DOI:** 10.1038/srep16713

**Published:** 2015-11-18

**Authors:** Da-Lu Liu, Na Lu, Wen-Juan Han, Rong-Gui Chen, Rui Cong, Rou-Gang Xie, Yu-Fei Zhang, Wei-Wei Kong, San-Jue Hu, Ceng Luo

**Affiliations:** 1 Institute of Neurosciences and Collaborative Innovation Center for Brain Science, Fourth Military Medical University, Xi’an 710032, China; 2 Department of Orthopedics, Xijing Hospital, Fourth Military Medical University, Xi’an 710032, China; 3 School of Clinical Medicine, Fourth Military Medical University, Xi’an 710032, China; 4 Department of Anesthesiology, Second Affiliated Hospital, Wenzhou Medical University, Wenzhou, 325027, China

## Abstract

Cervical radiculopathy represents aberrant mechanical hypersensitivity. Primary sensory neuron’s ability to sense mechanical force forms mechanotransduction. However, whether this property undergoes activity-dependent plastic changes and underlies mechanical hypersensitivity associated with cervical radiculopathic pain (CRP) is not clear. Here we show a new CRP model producing stable mechanical compression of dorsal root ganglion (DRG), which induces dramatic behavioral mechanical hypersensitivity. Amongst nociceptive DRG neurons, a mechanically sensitive neuron, isolectin B4 negative Aδ-type (IB4^−^ Aδ) DRG neuron displays spontaneous activity with hyperexcitability after chronic compression of cervical DRGs. Focal mechanical stimulation on somata of IB4^-^ Aδ neuron induces abnormal hypersensitivity. Upregulated HCN1 and HCN3 channels and increased *I*_h_ current on this subset of primary nociceptors underlies the spontaneous activity together with neuronal mechanical hypersensitivity, which further contributes to the behavioral mechanical hypersensitivity associated with CRP. This study sheds new light on the functional plasticity of a specific subset of nociceptive DRG neurons to mechanical stimulation and reveals a novel mechanism that could underlie the mechanical hypersensitivity associated with cervical radiculopathy.

Cervical radiculopathy can be initiated by cervical spondylosis and intervertebral disc diseases with dorsal root ganglion (DRG) affected in patients^1^. Pain is a typical symptom annoying the lives of 97.5% patients with cervical radiculopathy, which is more frequent than motor dysfunction and extremely difficult to treat^2^,^3^. Despite the high prevalence of cervical radiculopathy, most of our knowledge about radiculopathic pain has been obtained from studies on lumbar spine. However, given the different location and function, cervical radiculopathic pain cannot simply be assumed as the similar versions of the same cascades to the pain occurred in the lumbar spine. Although current studies have attempted to investigate cervical pain symptoms by employing transient cervical nerve root compression or crush, and brachial plexus avulsion^4^,^5^,^6^, these models may not reflect actual pathophysiological processes of radiculopathic pain resulting from a chronic compression of cervical DRGs or its near nerve root seen in clinic. It is therefore necessary and important to establish a new cervical radiculopathic pain model in animals which mimics the pain symptoms observed in human.

Clinically, mechanical hypersensitivity always associates with cervical radiculopathic pain (CRP), particularly because cervical DRGs are at higher mechanical risk for compression resulting from vertebral injuries, intervertebral disc herniation or intervertebral foramen stenosis during cervical vertebral motions^7^,^8^. However, the precise mechanisms underlying mechanical hypersensitivity of CRP are poorly understood. Sensitization of C and Aδ primary afferent nociceptors has been involved in mediation of neuropathic pain^9^. Binding to isolectin-B4 (IB4) have been used to define different subgroups of nociceptive dorsal root ganglion (DRG) neurons. C-fiber nociceptive DRG neurons can be divided into IB4-positive (IB4^+^) and IB4-negative (IB4^−^) subsets, while all Aδ-type nociceptive DRG neurons are IB4^−^ except some Aδ-low threshold mechanoreceptive (LTMs) neurons are weakly IB4^+ ^10. These neurochemical differences in discrete subsets of nociceptive DRG neurons are paralleled by different anatomical pathways and distinct electrophysiological characteristics as well as modality-specific contributions to the processing of pain messages^10^,^11^,^12^. Compelling evidence has revealed mechanically-activated currents or firing in the somata of DRG neurons including nociceptive DRG neurons that displayed distinct properties between different subsets of cells, a presumed basis for mechanotransduction of primary afferents^13^,^14^,^15^. Therefore, we are interested to know whether and which specific subset of nociceptive DRG neurons showed increased sensitivity to mechanical stimulation under cervical radiculopathy. Furthermore, what candidates are involved in the increased neuronal mechanical sensitivity, which in turn might result in the behavioral mechanical hypersensitivity associated with CRP?

To address the above questions, here we mimic CRP in patients by producing a new animal model with chronic compression of C7/C8 DRGs through insertion of a fine stainless steel rod into C7/C8 intervertebral foramen. The affected forepaw displayed a pronounced, long-lasting mechanical hypersensitivity (hyperalgesia and allodynia) that mimics the pain symptoms observed clinically. In addition, less dramatic but significant thermal hyperalgesia and frequent spontaneous pain behaviors were observed as well. On this model, we demonstrated that amongst nociceptive DRG neurons, IB4^−^ Aδ-fiber neurons but not IB4^+^ or IB4^−^ C-fiber neurons derived from CRP rats specifically exhibited hyperexcitability with persistent spontaneous discharges. Most importantly, this subset of DRG neurons is associated with hypersensitivity to focal mechanical stimulation in CRP rats. Upregulated HCN1 and HCN3 channels and increased *I*_h_ current on this subset of primary nociceptors is responsible for neuronal hypersensitivity to mechanical stimulation and thus behavioral mechanical hypersensitivity observed in CRP rats. This study sheds new light on the functional plasticity of a specific subset of nociceptive DRG neurons to mechanical stimulation and reveals a novel mechanism that could underlie the mechanical hypersensitivity associated with cervical radiculopathy.

## Results

### Mechanical hypersensitivity and thermal hyperalgesia in rats subjected to CRP

**Figure 1:**
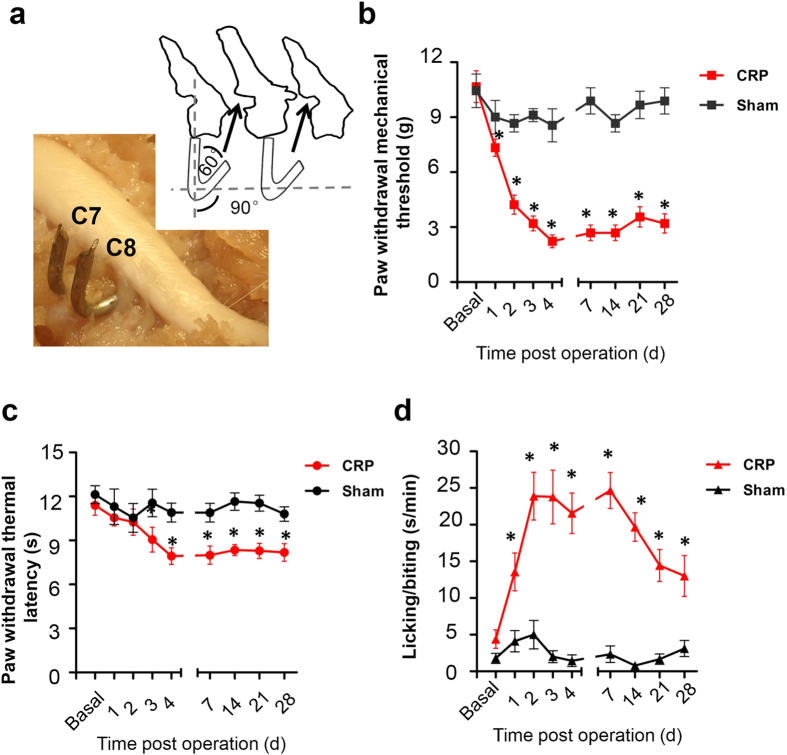
Setup of cervical radiculopathic pain (CRP) model following chronic compression of C7/C8 DRGs. (**a**) Schematic illustration of the method for producing a chronic compression of the C7/C8 DRGs in rat. Position and direction of a stainless steel rod inserted into the intervertebral foramen are shown. (**b**) Magnitude and time course of mechanical hypersensitivity to plantar von Frey hair application are shown in CRP and sham control rats. Note that paw withdrawal mechanical threshold decreased significantly from the 1st day after chronic compression and remained so up to the latest time point tested as compared to controls. (**c**) Showing the magnitude and time course of thermal hyperalgesia to radiant heat in CRP rats. Note that paw withdrawal thermal latency was significantly reduced till 3 days after operation, as compared to controls. (**d**) Spontaneous pain developed in CRP rats, as compared to controls. All data are expressed as mean ± S.E.M. *P < 0.05 as compared to their basal levels using repeated measures one-way analysis of variance (ANOVA).

Following chronic compression of C7/C8 DRGs (Fig. 1a), all the animals appeared in good health and did not show any signs of autotomy. Sensitivity of compressed rats to mechanical and thermal stimuli was tested at different time points post operation. Compared to control rats (n = 9), compressed rats showed a sharp drop in response threshold to Von Frey hairs in ipsilateral forepaws, reflecting mechanical hypersensitivity (Fig. 1b, n = 9, *P* < 0.001). This mechanical hypersensitivity started on the 1st day post operation, persisting all the way up to the latest time point tested, namely 28 d following operation. In contrast, thermal hyperalgesia to noxious plantar heat stimuli developed slowly and moderately in compressed rats (Fig. 1c, n = 9, *P* < 0.001). Until 3 days after operation, rats began to display significant decease in paw withdrawal latency. This thermal hyperalgesia has a much lesser extent compared to that found in inflammatory state^16^.

In addition, frequent spontaneous pain was observed at 1d after operation, manifesting as lifting, licking and biting the injured forepaw. From 2 to 14 d after surgery, the duration of spontaneous pain reached ~5.43-fold as that of basal level (Fig. 1d, n = 9, *P* < 0.001). Previous clinical studies have reported that moving the head away from the symptomatic side often improves CRP, whereas bending it toward the symptomatic side increases CRP^1^,^17^. Similar to patients, CRP rats also tend to bend the head toward the uninjured side, probably to relieve some pain (data not shown, n = 9).

### Increased c-Fos expression and ERK1/2 phosphorylation in DRG and spinal dorsal horn neurons in CRP rats

**Figure 2:**
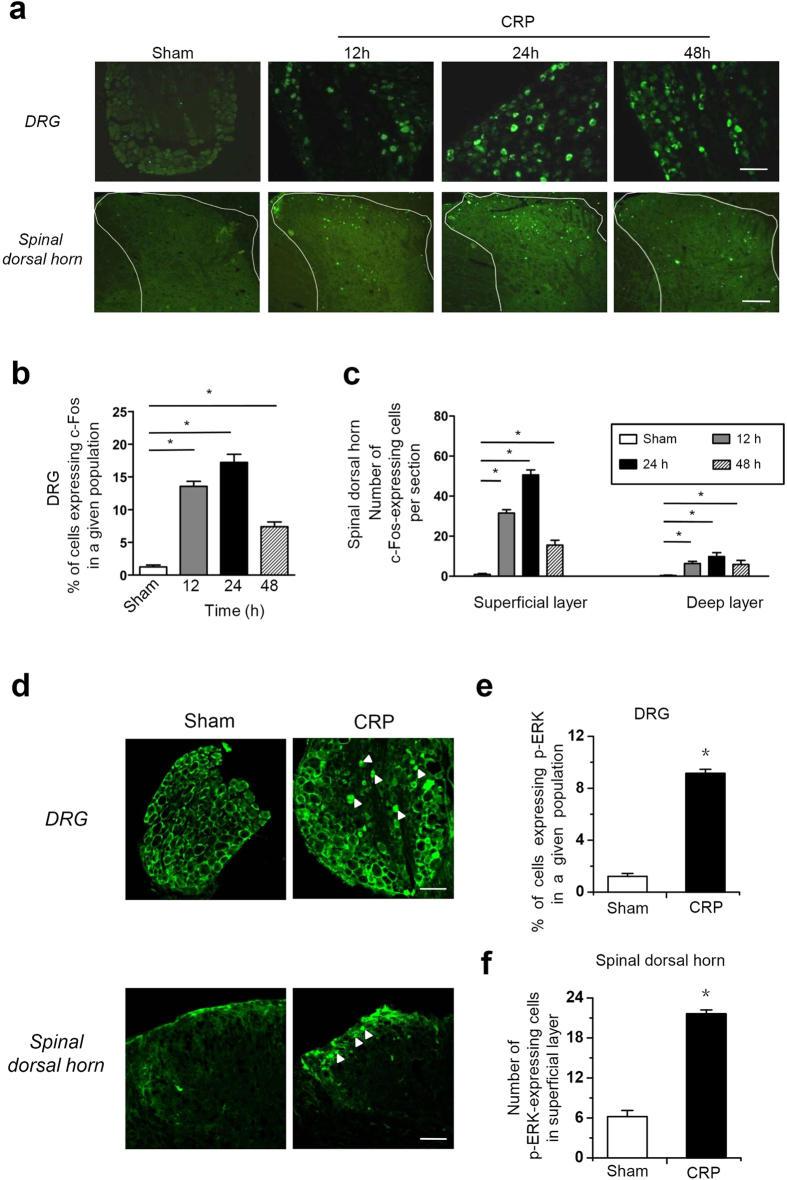
Induction of c-Fos expression and ERK1/2 phosphorylation in the DRG and spinal dorsal horn of the pain pathway following chronic compression of C7/C8 DRGs. (**a**) Typical examples of immunoreactivity for c-Fos in the DRG (upper panels) and spinal dorsal horn (lower panels) from control rats and CRP rats at 12 h, 24 h and 48 h post operation. (**b**) Quantification of the number of Fos-positive small cells in the DRG derived from control and CRP rats at different time points (n = 3 rats per group). (**c**) Quantification of the number of Fos-positive cells in the superficial and deep layer of spinal dorsal horn from control and CRP rats at different time points (n = 3 rats per group). (**d**) Typical examples of immunoreactivity for phosphorylated ERK1/2 in the DRG (upper panels, white solid triangle) and spinal dorsal horn (lower panels, white solid triangle) derived from control rats and CRP rats at 12 h post operation. (**e**) Quantification of phosphorylated ERK1/2 in small DRG neurons from control and CRP rats (n = 3 rats per group). (**f**) Quantification of phosphorylated ERK1/2 in the superficial layer of dorsal horn from control and CRP rats (n = 3 rats per group). All data are expressed as mean ± S.E.M. *P < 0.05 indicates statistically significant differences between control and CRP rats (ANOVA). Scale bars represent 100 μm in (**a**) and upper panels in (**d**), 50 μm in the lower panels in (**d**).

The immediate early gene c-fos is rapidly activated to express protein c-Fos by noxious stimuli at multiple avenues in pain pathways and is considered a reliable indicator for the activation status of neurons^18^,^19^. To get understanding of a profile of populated neuronal activity involved in CRP, we assayed the spatial and temporal expression of c-Fos protein in the DRG and spinal dorsal horn following compression of C7/C8 DRGs. As shown in Fig. 2a, compression of C7/C8 DRGs produced a significant upregulation of c-Fos expression in the ipsilateral DRG and spinal dorsal horn (Fig. 2, n = 3 rats). The number of Fos-positive small DRG neurons increased 10-fold over basal level at 12 h, reaching peak at 24 h and gradually declining at 48 h post operation (Fig. 2a,b, n = 3 rats). In the spinal dorsal horn, Fos-immunoreactivity began to appear at 12 h after operation, reaching peak in superficial (I–II) and deep (IV–V) lamina of spinal dorsal horn at 24 h after operation, then declining to a very low level after 48 h (Fig. 2a,c, n = 3 rats).

To further reveal the relevance of CRP model with nociceptive processing, we determined the activity-induced phosphorylation of the extracellular receptor-activated MAP Kinases 1/2 (ERK1/2) in the DRG and spinal cord. ERK1/2 are known to be selectively activated by peripheral nociceptive input and contribute to pain hypersensitivity^20^,^21^. As shown in Fig. 2d, we found that chronic compression of C7/C8 DRGs led to an intense increase of phosphorylation of ERK1/2 (pERK1/2) in small-diameter neurons of the compressed DRGs, as compared to sham group (see upper panels of Fig. 2d for typical examples and Fig. 2e for quantitative summary, *P* < 0.05, n = 3 rats). In the spinal cord, increased pERK1/2 induction was seen in the soma and neuropil of spinal lamina I upon DRGs compression, whereas the deeper lamina did not show any increase of pERK1/2 (see lower panels of Fig. 2d for typical examples and Fig. 2f for quantitative summary, *P* < 0.05, n = 3 rats). Taken together, these results indicate that chronic compression of cervical DRGs produced dramatic increased neuronal activity in the DRG and spinal dorsal horn, which further revealed the validity of CRP model as a reliable pain model.

### Changes of membrane properties and increased excitability in small DRG neurons derived from CRP rats

**Figure 3:**
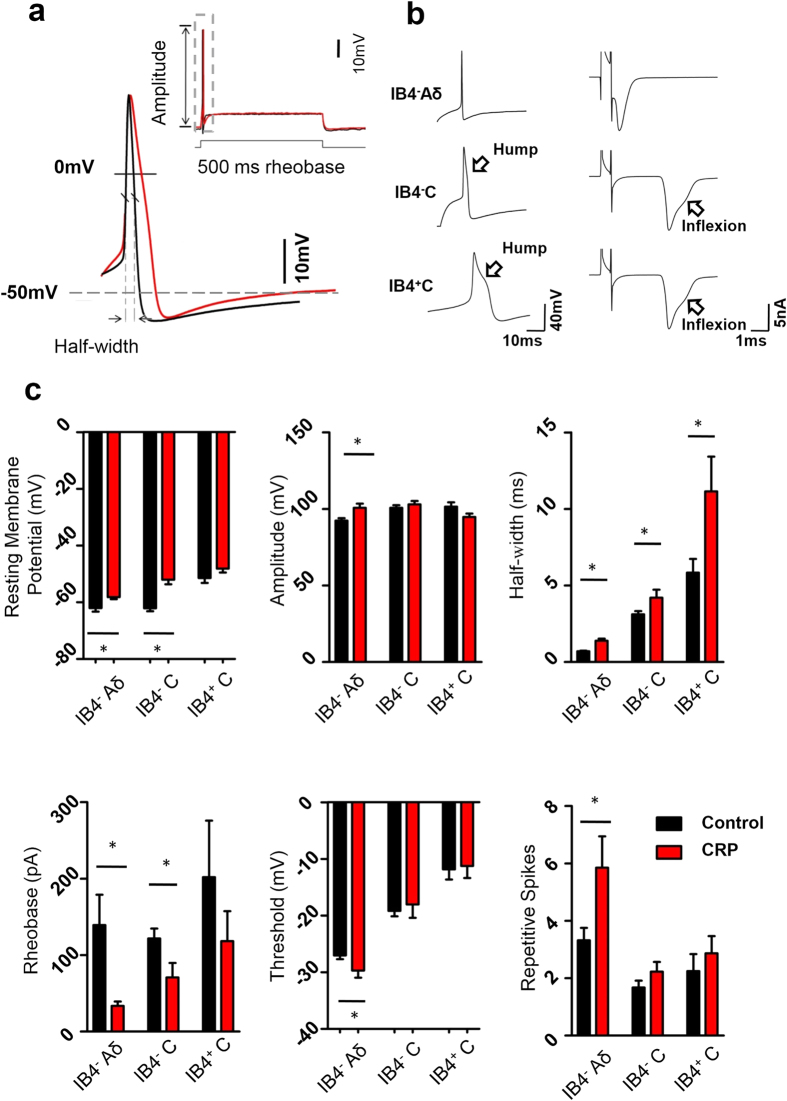
Membrane property and cell excitability of different subsets of nociceptive DRG neurons in control and CRP rats. (**a**) A representative action potential of whole-cell configuration evoked by a depolarizing current injection from control (in black) and from CRP (in red) group. (**b**) Classification of all small DRG neurons recorded. Note that three subtypes were determined by IB4 staining, shape of action potential (left panels) and type of the afferent. A single electrical stimulation of dorsal root 3 mm away from DRG was applied to distinguish C type afferent from Aδ type. (**c**) Showing resting membrane potential (RMP), action potential (AP) properties, such as amplitude, half-width, rheobase and threshold as well as repetitive firing in response to current injection in IB4^−^ Aδ-, IB4^−^ C- and IB4^+^ C-type DRG neurons from control and CRP rats. All data are expressed as mean ± S.E.M. *P < 0.05 indicates statistically significant differences between control and CRP rats (ANOVA).

To further investigate which subtypes of neurons were involved in the development of CRP, a total of 213 small-diameter nociceptive DRG neurons (≤ 25 μm) without any spontaneous property from 47 rats were tested for their membrane properties and excitability, 111 neurons from 26 control rats and 103 from 21 CRP rats (Fig. 3a). The recorded small DRG neurons were classified by both the conduction velocity (CV) and IB4 binding. Neurons with CV less than 0.8 m/s were classified as C-type nociceptive neurons, which can be further divided into two subsets by IB4 binding, namely IB4-positive C-fiber neurons (IB4^+^ C) and IB4-negative C-fiber neurons (IB4^−^ C). Neurons with CV at 1.5–6.5 m/s were classified as Aδ-fiber nociceptive neurons, which are IB4-negative (IB4^−^ Aδ). A few Aδ neurons with weak IB4 binding are not included in our study since it has been reported to be Aδ-low threshold mechanoreceptive neurons^10^. Consistent with previous reports^10^, C-fiber neurons have significantly longer action potential (AP) duration than Aδ-fiber neurons, which is due to the inflexion (hump) on the falling phase (Fig. 3a,b). Amongst C-fiber neurons, AP duration in IB4^+^ C neurons is much longer than that in IB4^−^ C neurons (Fig. 3b,c).

Following compression of C7/C8 DRGs, IB4^−^ Aδ neurons exhibited dramatic changes in membrane properties and enhanced excitability. For instance, compressed IB4^−^ Aδ neurons (Fig. 3c, n = 35 *vs* 25 neurons from 7 CRP *vs* 7 control rats, *P* = 0.002) get more depolarized in resting membrane potential, with significant reduction in input resistance (146.4 ± 55.7 MΩ) when compared to control neurons (233.8 ± 32.6 MΩ, *P* = 0.02, n = 12 *vs* 10 neurons from 7 CRP *vs* 7 control rats). Membrane capacitance in IB4^−^ Aδ neurons was not different between two groups (34.6 ± 2.3 pF *vs* 39.0 ± 2.2 pF in CRP *vs* control rats) (*P* > 0.05, n = 18 *vs* 18 neurons from 7 CRP *vs* 7 control rats). Enhanced excitability in IB4^−^ Aδ neurons from CRP rats is manifested as increased AP amplitude, prolonged AP half-width, lowered AP threshold as well as reduced rheobase (Fig. 3c, n = 35 *vs* 25 neurons from 7 CRP *vs* 7 control rats, *P* < 0.05). Given the depolarized RMP and lowered AP threshold in compressed IB4^−^ Aδ DRG neurons, the voltage difference (ΔV) from RMP to the threshold required to evoke AP in compressed DRG neurons is much decreased in comparison with control ones. Even though input resistance or rheobase or both is reduced, it is still enough to excite the neuron to fire AP. Based on this, it is reasonable that increased excitability in compressed IB4^−^ Aδ DRG neurons displayed a reduction in input resistance. In contrast, IB4^−^ and IB4^+^ C neurons inconsistently showed changes in only a few AP variables after chronic compression (Fig. 3c). More importantly, the mean firing frequency induced by a depolarizing current step was much higher in IB4^−^ Aδ neurons (Fig. 3c, n = 11 *vs* 10 neurons from 7 CRP *vs* 7 control rats, *P* = 0.038), but not in IB4^−^ or IB4^+^ C neurons derived from CRP rats than that from control rats. These results indicate that hyperexcitability of IB4^−^ Aδ-fiber DRG neurons might be closely related to the development of CRP.

### High incidence of ongoing spontaneous firing in IB4^−^ Aδ-fiber neurons of CRP rats

**Figure 4:**
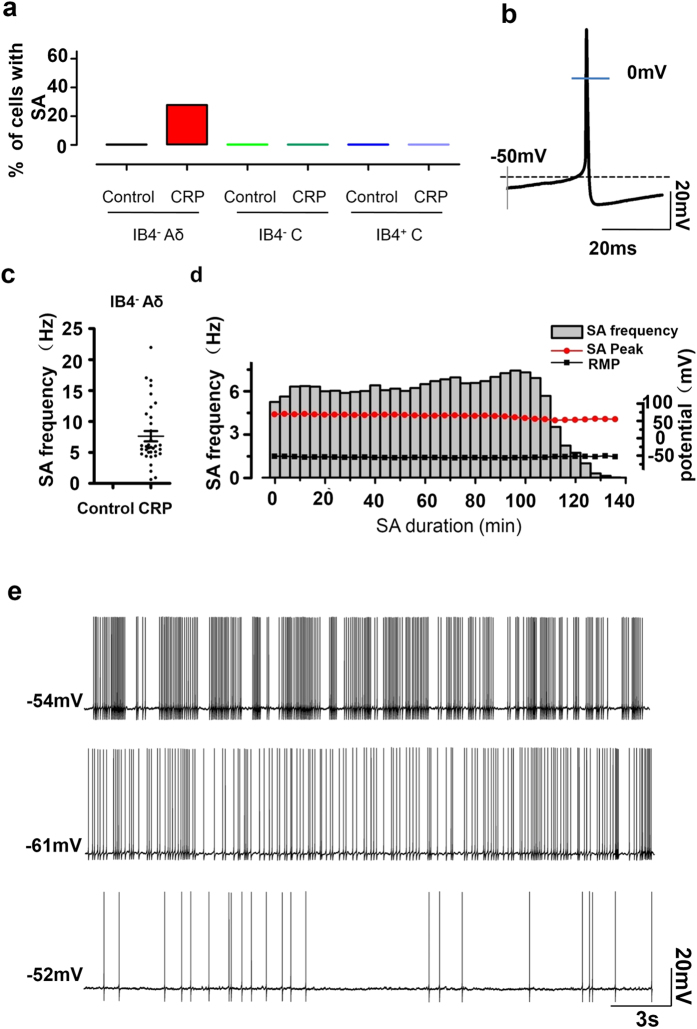
Occurrence of spontaneous activity (SA) in different subsets of nociceptive DRG neurons in CRP rats. (**a**) The proportion of cells expressing SA in IB4^−^ Aδ-, IB4^−^ C- or IB4^+^ C-type DRG neurons in control and CRP rats. (**b**) Typical example of a spontaneous firing of an IB4^−^ Aδ-type neuron from CRP rats at 3d after surgery. (**c**) Quantitative analysis of SA frequency in IB4^−^ Aδ-type neurons in control and CRP rats. (**d**) The time course of a typical example of spontaneous firing observed in IB4^−^ Aδ-type neuron from CRP rats showing frequency (grey column), peak amplitude (filled red circle), RMP (filled black rectangle) and duration of SA. (**e**) Three firing patterns of SA in IB4^−^ Aδ-type neuron from CRP rats are shown: bursting activity (upper panel), periodic discharges (middle panel) and irregular firing (lower panel). *P < 0.05 indicates statistically significant differences between control and CRP rats (ANOVA).

Previous studies have reported that the injured lumbar DRG neurons represented ectopic spontaneous activity with single fiber recording, most of the fibers were assumed to be Aβ type^22^,^23^. In the present study, we observed that frequent spontaneous firing was seen in 28% (35/126) of IB4^−^ Aδ-fiber DRG neurons upon testing at 3 d after the onset of chronic compression. In striking contrast, none of compressed IB4^−^ C or IB4^+^ C neurons were spontaneously active (Fig. 4a,b). Of the total 119 small DRG neurons tested from normal C7/C8 DRGs, none of them (0%) showed spontaneous activity. Neurons with spontaneous firing were excluded if the resting membrane potential >−45 mV or AP peak <0 mV (Fig. 4b). Quantitative analysis revealed that the mean frequency of spontaneous firing reached 7.6 ± 0.8 Hz (Fig. 4c, n = 35 neurons from 7 rats). This spontaneous activity can last for ~2 hours until whole-cell configuration was unstable and lost (Fig. 4d). Three different firing patterns have been previously reported in the injured lumbar A-fiber DRG neurons according to the dynamic features of interspike interval series, that is periodic, non-periodic (irregular) and bursting activity^22^. In our case, most patterns of spontaneous firing (29/35) fell into bursting activity with intermittences during continuous spikes (Fig. 4e, upper panel). Four out of 35 neurons displayed periodic discharges with high-frequency (>15 Hz) (Fig. 4e, middle panel), only 2 out of 35 was found to fire irregularly at very low frequency (1 HZ or lower) (Fig. 4e, lower panel).

### Hypersensitivity of IB4^−^ Aδ-fiber DRG neuron somata to focal mechanical stimulation in CRP rats

**Figure 5:**
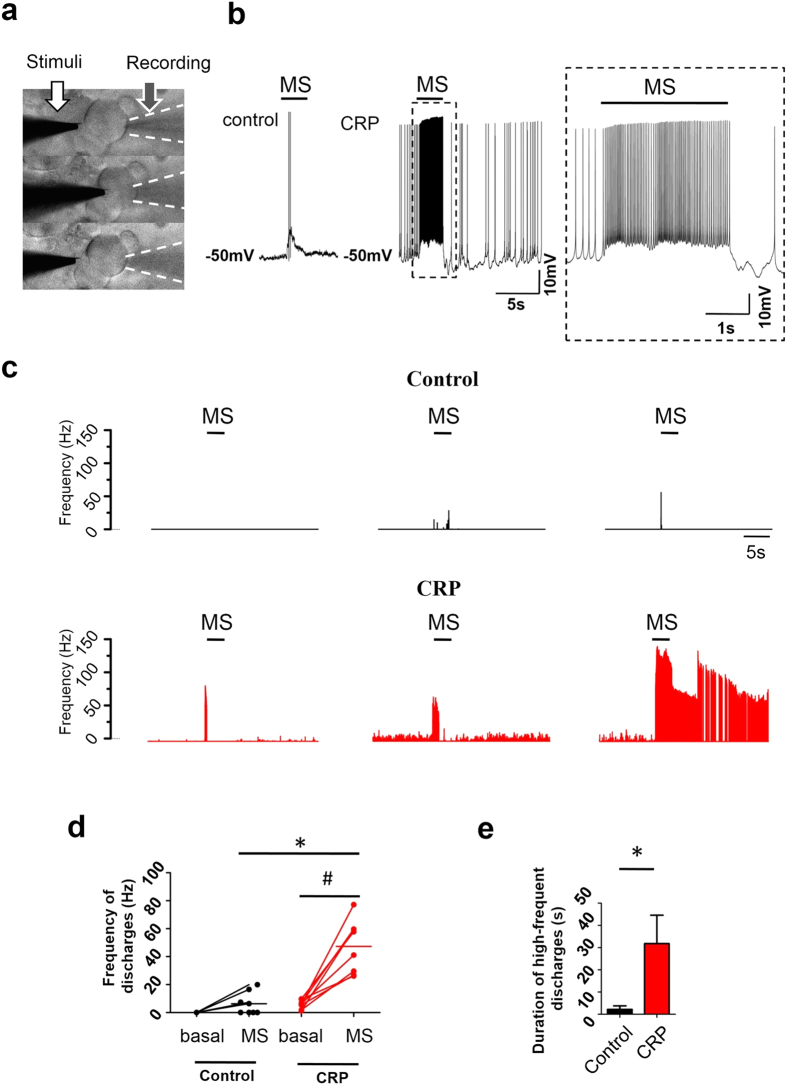
Hypersensitivity to focal mechanical stimulation in IB4^−^ Aδ type neurons from CRP rats. (**a**) Focal mechanical stimulation was applied to the somata of IB4^−^ Aδ neurons using a slow-moving heat-polished glass pipette (white arrow). Membrane deflection was observable by every stimulation (about 1/6 diameter of the neuron, 5–7 μm) for 1 s in duration. Response of the cell was recorded by a recording pipette (black arrow). (**b**) A typical example of the mechanical response in IB4^−^ Aδ neuron from control and CRP rats. Inset is a magnification of response from CRP DRG neurons. (**c**) Representative examples of mechanically activated property of 3 different IB4^−^ Aδ neurons from control and CRP group, respectively. Note that long-lasting afterdischarge was seen in 62.5% (5 out of 8) of DRG neurons tested in CRP group. (**d**) Firing frequency evoked by mechanical stimulation was much stronger in IB4^−^ Aδ neurons from CRP rats as compared to that from control rats. (**e**) Quantitative analysis of duration of the high-frequent discharges to mechanical stimulation in control and CRP group. The durations were determined from the onset of the high-frequent discharges until spike frequency returned to pre-stimulus level. MS: mechanical stimulation. All data are expressed as mean ± S.E.M. *P < 0.05 as compared to control rats and ^#^*P* < 0.05 as compared to basal level (ANOVA).

It has been reported that DRG neuron somata respond to mechanical stimulation, with more IB4^−^ DRG neurons expressing larger slowly adapting mechanosensitive inward current than IB4^+^ neurons^14^,^24^. Given the above observation that IB4^−^ Aδ neurons became hyperexcitable upon chronic compression and compressed rats displayed mechanical hyperalgesia and allodynia, we further asked whether injured IB4^−^ Aδ neurons exhibit hypersensitivity to mechanical stimulation. As shown in Fig. 5, upon application of focal mechanical stimulation to the somata (Fig. 5a), IB4^−^ Aδ neurons from CRP rats showed much stronger response to mechanical stimulation than that derived from control rats (representative traces in Fig. 5b; frequency histogram in Fig. 5c). The mean firing frequency evoked by the same mechanical stimulation in CRP IB4^−^ Aδ neurons was much higher (47.3 ± 6.7 Hz) than that in control neurons (5.0 ± 2.4 Hz) (Fig. 5d, n = 8 *vs* 10 neurons from 6 CRP *vs* 6 control rats, *P* < 0.05). In addition, most of the injured neurons (5/8) displayed long-lasting afterdischarge after the stimulus was ceased, which was not seen in control neurons (Fig. 5c). Analysis of the duration of mechanical response revealed an 18.4-fold increase in injured IB4^−^ Aδ neurons compared to control ones (Fig. 5e, n = 8 *vs* 10 neurons from 6 CRP *vs* 6 control rats, *P* = 0.018).

### Upregulation of *I*_h_ in IB4^−^ Aδ-fiber neurons from CRP rats

**Figure 6:**
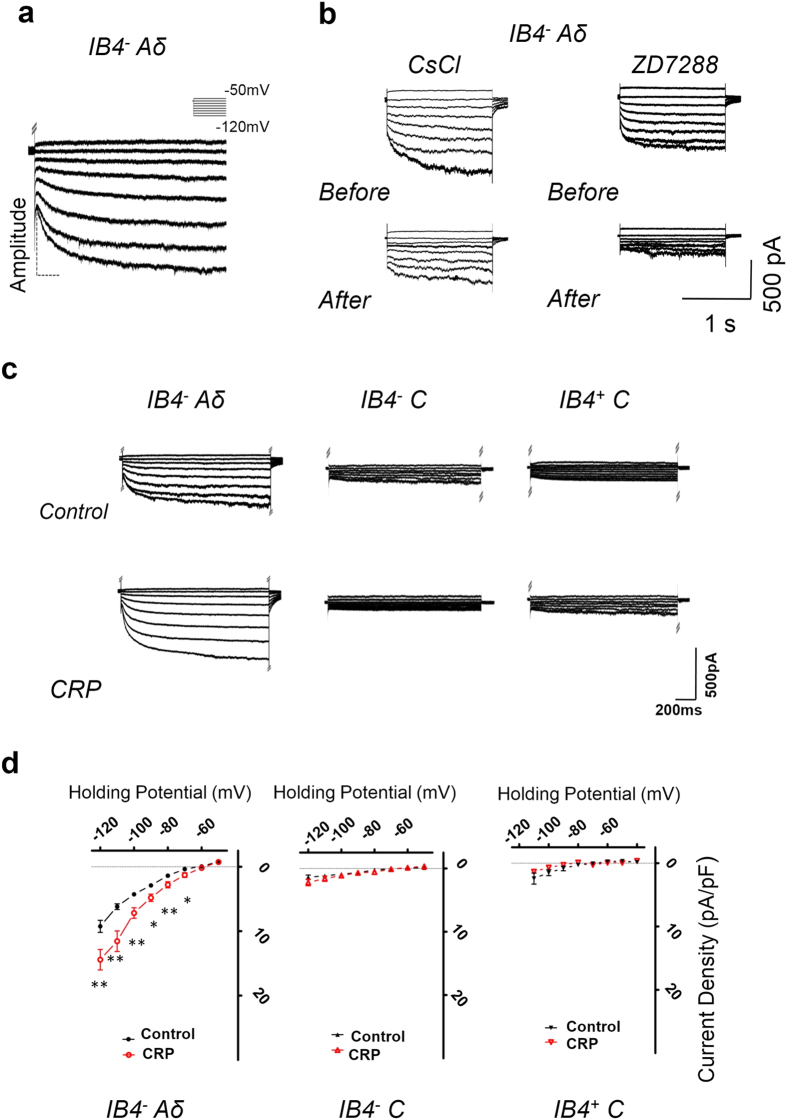
CRP increases *I*_h_ currents in IB4^−^ Aδ-, but not in IB4^−^ C- or IB4^+^ C-type DRG neurons. (**a**) *I*_h_ current was evoked by hyperpolarizing voltage steps of −50 mV to −120 mV from a holding potential at −60 mV in IB4^−^ Aδ-type DRG neurons. The voltage protocol is shown at the top. (**b**) *I*_h_ current recorded in IB4^−^ Aδ-type DRG neurons was blocked by bath application of Cs^2+^ (1 mM) or ZD7288 (15 μM). (**c**) Representative examples of *I*_h_ recorded in IB4^−^ Aδ-, IB4^−^ C- and IB4^+^ C-type DRG neurons from control (upper panels) and CRP rats (lower panels). (**d**) Only IB4^−^ Aδ-type neurons showed upregulated *I*_h_ currents after CRP operation, which is not seen in C-type neurons. All data are expressed as mean ± S.E.M. *P < 0.05 as compared to control rats.

Numerous studies have provided evidence that hyperpolarization-activated cation current (*I*_h_) is important in influencing the excitability and driving repetitive firing in primary nociceptive neurons and pain pathophysiology^25^,^26^,^27^,^28^. Here we are interested to know whether *I*_h_ expression is altered in different types of nociceptive DRG neurons after chronic compression of C7/C8 DRGs and whether it is involved in the hyperexcitability of IB4^−^ Aδ-fiber neurons and its mechanical hypersensitivity in the development of CRP. Consistent with previous reports^29^,^30^, *I*_h_ recorded in our study had the following characteristics (Fig. 6a,b): (1) an inward current in response to hyperpolarizing potentials of −50 to −120 mV from a holding potential of −60 mV (Fig. 6a); (2) highly sensitive to monovalent ion Cs^+^ (1 mM) or *I*_h_-selective antagonist, ZD7288 (15 μM) (Fig. 6b). Although inward rectifier K^+^ current could also be evoked by these hyperpolarizing voltage steps, this current is unlikely to contribute to the recorded current because of its fast, instantaneous activation kinetics and very low expression in DRG neurons^31^.

Step hyperpolarization from −50 mV to −120 mV resulted in slowly inward currents in control IB4^−^ Aδ, IB4^−^ C as well as IB4^+^ C-fiber neurons. In general, Aδ neurons displayed a greater magnitude of *I*_h_ than C neurons (Fig. 6c). Figure 6d shows *I*-*V* relations of *I*_h_ in three different types of nociceptive DRG neurons derived from CRP and control rats. When normalized to cell capacitance, the median *I*_h_ current density in IB4^−^ Aδ neurons from CRP rats was significantly increased compared to that from control rats (Fig. 6d, left panel, n = 13 *vs* 17 neurons from 6 CRP *vs* 6 control rats, *P* < 0.05). In contrast, IB4^−^ C or IB4^+^ C neurons displayed no obvious alterations in *I*_h_ current density after chronic compression (Fig. 6d, middle panel for IB4^−^ C neurons, n = 17 *vs* 7 neurons from 6 CRP *vs* 6 control rats, *P* > 0.05; right panel for IB4^+^ C neurons, n = 11 *vs* 9 neurons from 6 CRP *vs* 6 control rats, *P* > 0.05). Amongst IB4^−^ Aδ neurons from CRP rats, very similar *I*_h_ magnitude was seen in neurons with and without spontaneous firing, so we pooled these two lines of *I*_h_ together.

**Figure 7:**
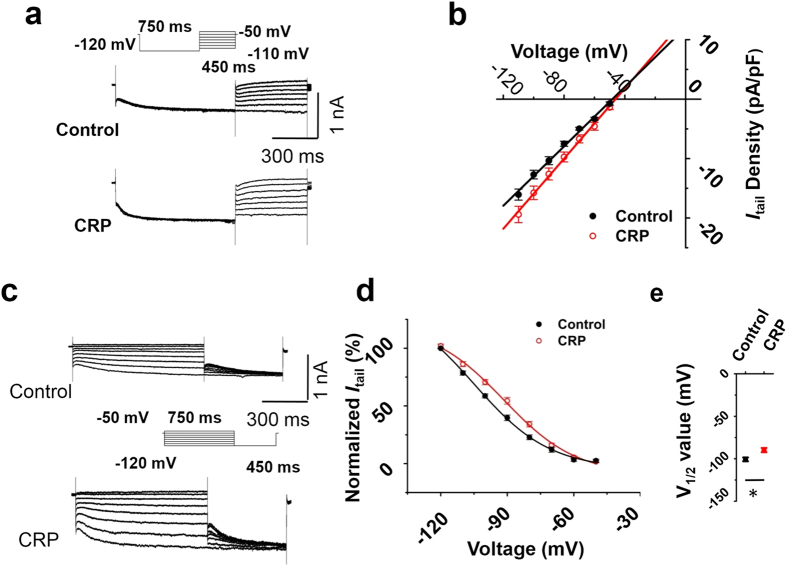
CRP changed voltage dependence of *I*_h_ activation in IB4^−^ Aδ type neuron, but not the reversal potential. (**a**) Reversal potential of *I*_h_ from control (middle panel) and CRP IB4^−^ Aδ type neurons (lower panel) were achieved by first applying a prepulse to −120 mV to fully activate *I*_h_ and then examining the tail currents after repolarization to test potentials from −110 to −50 mV (upper panel). Tail currents were plotted against test potentials. (**b**) No difference of reversal potential of *I*_h_ current was shown between control (black) and CRP (red) group. (**c**) The activation curve of *I*_h_ from control (upper panel) and CRP IB4^−^ Aδ neurons (lower panel) was constructed by measuring tail currents at −120 mV after application of prepulse potentials between −50 to −120 mV (middle inset) and fitted with a Boltzmann equation. (**d**) The midpoint (V_1/2_) for activation of *I*_h_ in the CRP IB4^−^ Aδ neurons (red) was shifted 11 mV in the depolarizing direction compared to control neurons (black). (**e**) The difference of V_1/2_ for activation of *I*_h_ between two groups was significant. All data are expressed as mean ± S.E.M. *P < 0.05 as compared to control rats.

Determination of the reversal potential of *I*_h_ were achieved by first applying a prepulse to −120 mV to fully activate *I*_h_ and then examining the tail currents after repolarization to test potentials from −110 to −50 mV (Fig. 7a). Plot of tail currents against test potentials revealed no significant alterations in the reversal potential of *I*_h_ between CRP and control IB4^−^ Aδ DRG neurons (Fig. 7b, n = 13 *vs* 12 neurons from 6 CRP *vs* 6 control rats, *P* < 0.05). As expected from the data in Fig. 6d, CRP rats exhibited a marked enhancement in *I*_h_ conductance (*P* < 0.05).

Voltage-dependent activation and inactivation are physiologically important characteristics of ion channels that can directly influence the excitability of neurons. The activation curve of *I*_h_ was constructed by measuring tail currents at −120 mV after application of prepulse potentials between −50 to −120 mV and fitted with a Boltzmann equation (Fig. 7c). As shown in Fig. 7d, the midpoint (V_1/2_) for activation of *I*_h_ in the CRP IB4^−^ Aδ neurons (−89.8 ± 2.5 mV) was shifted 11 mV in the depolarizing direction compared to control neurons (−100.6 ± 2.3 mV). The difference of V_1/2_ for activation of *I*_h_ between two groups was significant (Fig. 7e, n = 16 *vs* 11 neurons from 6 CRP *vs* 6 control rats, *P* < 0.05). Taken together, chronic compression of C7/C8 DRGs produced an upregulation of *I*_h_ in nociceptive DRG neurons, particularly in subtypes of IB4^−^ Aδ neurons, which is different from that reported previously in inflammatory pain states^30^.

### Enhanced expression of HCN1 and HCN3, but not HCN2 in IB4^−^ small DRG neurons from CRP rats

**Figure 8:**
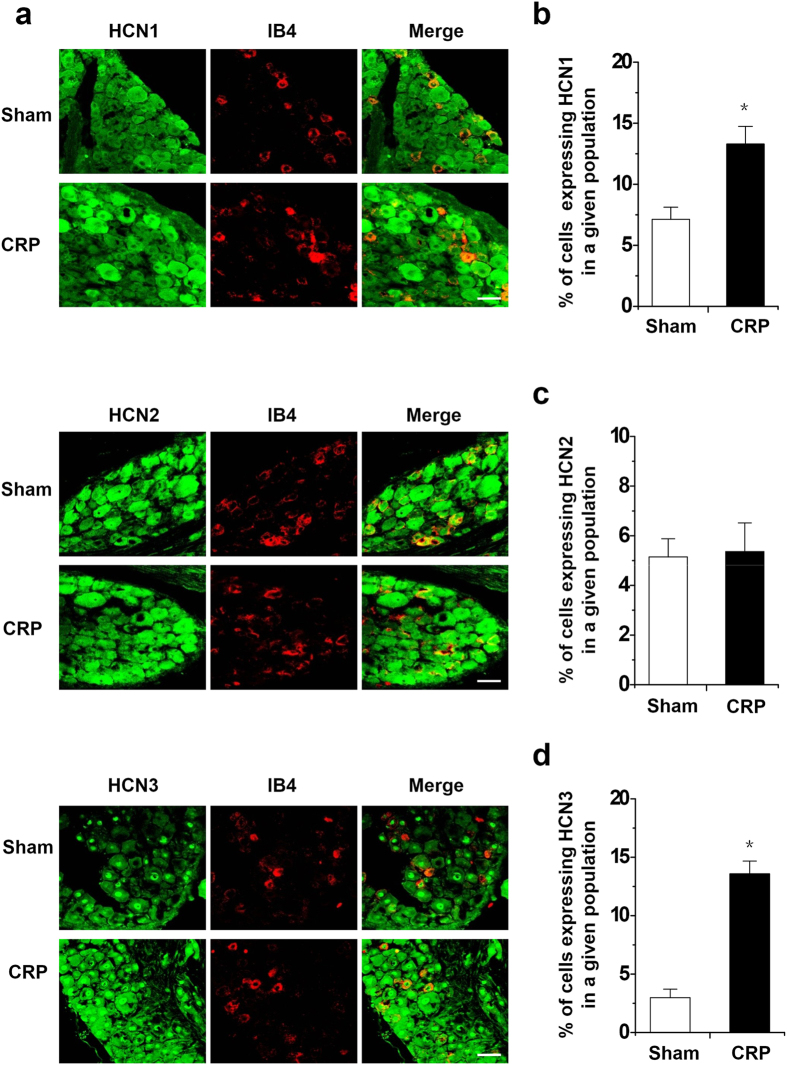
Increased expression of HCN1 and HCN3, but not HCN2 in IB4^−^ small diameter DRG neurons in CRP rats compared to controls. (**a**) Double immunofluorescence staining of DRG neurons with IB4 (red) and HCN1 (green, upper panels), HCN2 (green, middle panels), HCN3 antibody (green, lower panels) in CRP and control rats. (**b**,**d**) Quantitative summary from double immunofluorescence experiments showing that in CRP rats, HCN1 (**b**) and HCN3 (**d**) immunoreactivity in IB4^−^ small diameter DRG neurons was increased in comparison with control rats. The percentage of IB4^−^ small diameter DRG neurons expressing HCN1 immunoreactivity in all DRG neurons is shown. (**c**) HCN2 immunoreactivity in IB4^−^ small diameter DRG neurons did not show obvious alteration in CRP rats compared to controls. All data are represented as mean ± SEM. Scale bars represent 50 μm. *P < 0.05 as compared to control rats.

Having established that chronic C7/C8 DRGs compression-induced increase in excitability of IB4^−^ Aδ neurons is associated with increased *I*_h_ amplitude and density in these neurons, we sought to determine whether chronic cervical DRGs compression alters HCN channel protein expression in DRG neurons, particularly in IB4^−^ small-diameter neurons. We focused on HCN1–3 because these isoforms are clearly expressed in rat DRG neurons, whereas the expression of HCN4 is uncertain^32^. Consistent with that reported by Kouranova *et al*.^32^, HCN1 and HCN3 are both expressed in large- and small-diameter cervical DRG neurons, whereas HCN2 channels are mostly expressed in small-diameter DRG neurons. Following chronic compression of cervical DRGs, HCN1 channels display a dramatic upregulation in IB4^−^ small-diameter neurons, as compared to control group (see typical examples in the upper panels of Fig. 8a and quantitative summary in Fig. 8b).

Similar to the increased expression pattern of HCN1, HCN3 displayed an enhanced expression in IB4^−^ small DRG neurons from CRP rats as compared to controls (see typical examples in the lower panels of Fig. 8a and quantitative summary in Fig. 8d, *P* < 0.05, n = 3 rats). In contrast, HCN2 expression did not show obvious alteration in IB4^−^ small-diameter neurons after CRP surgery (see typical examples in the middle panels of Fig. 8a and quantitative summary in Fig. 8c, *P* > 0.05, n = 3 rats). This suggest that enhanced expression of HCN1 and HCN3 isoforms in IB4^−^ small-diameter neurons might contribute to upregulation of *I*_*h*_ current recorded from CRP rats.

### Both hypersensitivity of IB4^−^ Aδ type neurons to mechanical stimulation and behavioral mechanical hypersensitivity in CRP rats are *I*_h_ dependent

**Figure 9:**
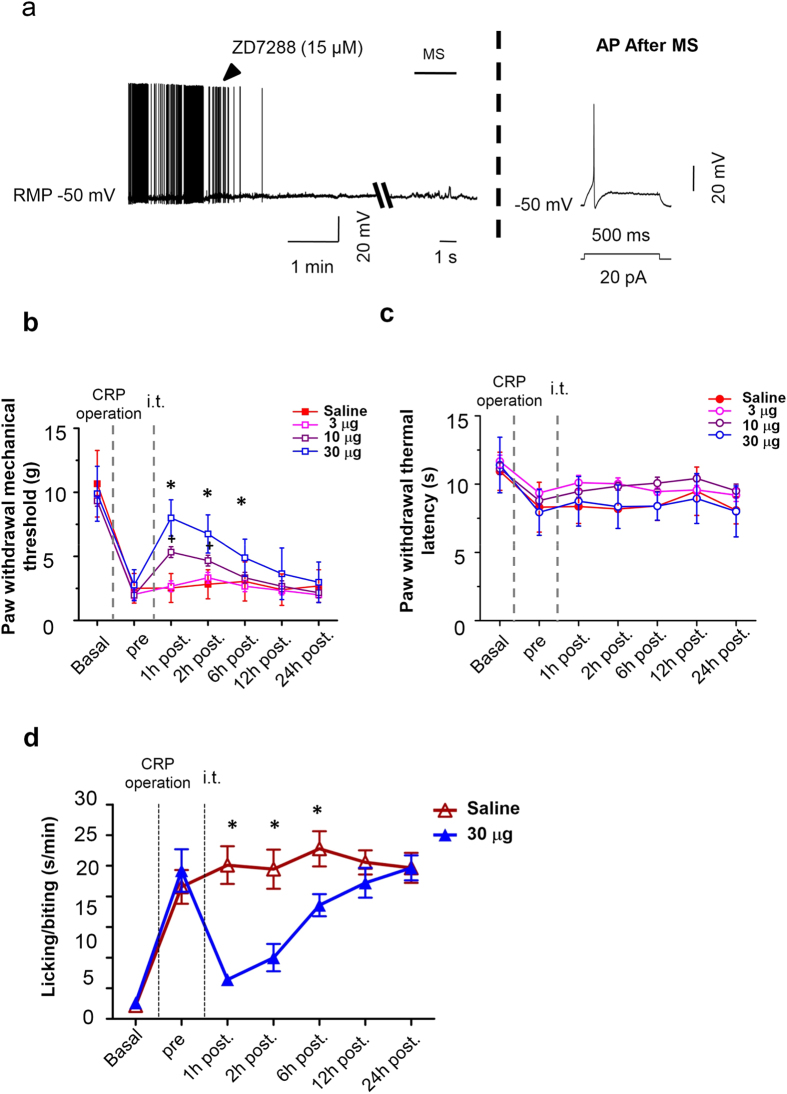
Spontaneous activity (SA) of IB4^−^ Aδ type neurons and its hypersensitivity to mechanical stimulation as well as behavioral mechanical hypersensitivity in CRP rats are *I*_h_ dependent. (**a**) ZD7288 blocked both SA and mechanical hypersensitivity of IB4^−^ Aδ type neurons from CRP group, without obvious influence of resting membrane potential (n = 3 neurons from 3 rats). Action potential was evoked after tests to determine whether the generation of action potential was compromised. (**b**,**c**) Time course of behavioral mechanical hypersensitivity (**b**) and thermal hyperalgesia (**c**) from CRP rats before (pre) and after i.t. injection of 3, 10, 30 μg ZD7288 or saline. Note that mechanical hypersensitivity was alleviated by ZD7288 dose-dependently, while thermal hyperalgesia was unaffected (n = 9–10 rats for each group). (**d**) Spontaneous pain from CRP rats was largely attenuated by ZD7288 as well (n = 9–10 rats for each group). All data are expressed as mean ± S.E.M. *P < 0.05 as compared to control group.

We next addressed whether upregulated *I*_h_ may contribute to the high incidence of spontaneous firing and mechanical hypersensitivity in IB4^−^ Aδ neurons after chronic compression of cervical DRGs. As shown in Fig. 9a, bath application of *I*_h_ selective antagonist, ZD7288 (15 μM) eliminated spontaneous firing in IB4^−^ Aδ neurons derived from CRP rats in a reversible manner (Fig. 9a, n = 3 neurons from 3 rats). The same concentration of the drug did not exert obvious effect on resting membrane potential. This is different from the obvious membrane hyperpolarization reported in other studies^33^. This difference might be due to different concentration used in our study with whole mount DRG preparation (15 μM) and in previous studies with brain slices (50 μM)^33^. More importantly, mechanical hypersensitivity of CRP IB4^−^ Aδ neurons in response to focal mechanical stimulation could be abolished by ZD7288 as well (Fig. 9a, n = 3, *P* < 0.05). To further confirm the abolishment of mechanical stimuli-induced firing by ZD7288 is not due to the bad cellular condition, we applied depolarizing stimuli to the neuron to check if normal AP can be evoked at the end of mechanical stimulation. These results suggest that enhancement of *I*_h_ after chronic compression of cervical DRGs might contribute to an increased incidence of spontaneous activity and mechanical hypersensitivity in IB4^−^ Aδ neurons.

We then went on to address whether these findings bear relevance to pain-related behavior *in vivo*. Intrathecal administration has been shown to be a feasible way to affect DRG sensory neurons^34^,^35^. In our study, *I*_h_ selective antagonist, ZD7288 (3, 10, 30 μg) was intrathecally (i.t.) delivered at 3d after chronic compression of cervical DRGs when stable and remarkable hyperalgesia and allodynia was produced. As compared to vehicle-injected group, i.t. ZD7288 resulted in a significant increase of mechanical threshold to von Frey hairs in a dose-dependent manner (Fig. 9b, n = 9, *P* < 0.01), but not thermal latency to heat stimulation (Fig. 9c, n = 9, *P* > 0.05), indicative of attenuation of behavioral manifestation of mechanical hyperalgesia and allodynia, but not thermal hyperalgesia. This antinociceptive effect of ZD7288 in CRP rats was significant within 12 h of drug administration. In parallel, spontaneous pain observed in CRP rats was largely suppressed by ZD7288 as well (Fig. 9d, n = 9, *P* < 0.01). Consistent with our results, a previous study by Takasu *et al*. has reported a suppressive effect of i.t. ZD7288 on the mechanical allodynia induced by partial ligation of the sciatic nerve^36^. Together with the observation that increased expression of HCN1 and HCN3 channels occurs at IB4^−^ small DRG neurons after CRP operation, it inferred us that antihyperalgesic effect of i.t. ZD7288 might be mainly originating from affecting peripheral DRG neurons. Although an earlier report by Chaplan *et al*. did not observe an antihyperalgesic effect by i.t. ZD7288, a clear dose-response effect (1–10 mg/kg) was shown by intraperitoneal (i.p.) ZD7288, suggesting that the effect of ZD7288 was peripheral, but not central, in origin^25^. This suggestion is supported by our observation that the increase of HCN1 and HCN3 channels immunoreactivity is seen in the soma of IB4^−^ small DRG neurons, which lies outside the central nervous system. In conclusion, these results support an essential role for upregulation of *I*_h_ in IB4^−^ Aδ neurons in neuronal and behavioral mechanical hypersensitivity associated with cervical radiculopathy.

## Discussion

Despite the highly-increasing incidence of cervical radiculopathy, most of our knowledge about radiculopathic pain has been obtained from studies on lumbar spine. However, given the different location and function from lumbar spine, cervical radiculopathic pain cannot simply be assumed as the similar versions of the same cascades to the pain occurred in the lumbar spine. Although current studies have attempted to investigate cervical pain symptoms by employing either transient (15 min) cervical nerve root compression (or crush), or brachial plexus avulsion (or ligation)^4^,^5^,^6^, these models may not reflect actual pathophysiological processes of radiculopathic pain resulting from a chronic compression of cervical DRGs or its near nerve root seen in clinic. In the present study, we present a new method of producing a chronic compression of C7/C8 DRGs in rats by inserting a fine stainless steel rod into corresponding intervertebral foramen and thereby producing a stenosis of the intervertebral foramen. The affected forepaw displayed a pronounced, long-lasting behavioral phenotype characterized by intense mechanical hypersensitivity (hyperalgesia and allodynia) that mimics the pain symptoms observed clinically. In addition, significant thermal hyperalgesia and frequent spontaneous pain behaviors were observed as well. Wide expression of c-Fos protein and phosphorylation of ERK1/2 observed in important regions in pain pathways, small DRG neurons and nociceptive lamina of spinal dorsal horn after chronic DRG compression has further revealed the validity of this animal cervical radiculopathic pain model.

One of the most striking findings of the present study is that IB4^−^ Aδ-fiber nociceptive DRG neurons showed ongoing spontaneous firing and increased excitability after chronic compression of C7/C8 DRGs. Consistent with this result, ectopic spontaneous activity in lumbar A-fiber DRG neurons have been demonstrated under various pathological states, e.g. lumbar dorsal root ganglion compression^23^,^37^, chronic constriction injury of sciatic nerve^38^, peripheral or central axotomy^39^. However, specific types of A-fiber neurons involved were not clearly differentiated in these studies. Although some studies assumed that most of the fibers with ectopic spontaneous activity in the injured lumbar DRG neurons were Aβ type^22^,^23^, whether and how Aδ type DRG neurons is involved in radiculopathic pain has not been reported. To the best of our knowledge, this is the first study to demonstrate a crucial role of ectopic activity and hyperexcitability of Aδ type DRG neurons in the development of cervical radiculopathic pain. Although IB4^+^ or IB4^−^ C-fiber neurons did not show ectopic activity and consistent changes in excitability upon chronic cervical DRGs compression in our case, important roles of C type DRG neurons in the development of cervical radiculopathic pain cannot be excluded. Much evidence has revealed that C type nociceptors display hyperexcitability and are assumed to be involved in various neuropathic pain states^40^,^41^,^42^. In striking contrast to the ectopic spontaneous activity in IB4^−^ Aδ nociceptors under cervical radiculopathic states, CFA-induced inflammatory states are associated with increased spontaneous activity in C but not Aδ-nociceptors^30^. This discrepancy suggests that cervical radiculopathic pain may activate a different pathway, which involves activation of different classes of nociceptors from inflammatory pain. A causal link has been revealed between spontaneous afferent firing and the development of spontaneous pain, allodynia and hyperalgesia^43^,^44^. Thus, increased spontaneous activity together with hyperexcitability in IB4^−^ Aδ-fiber nociceptive DRG neurons may result in a substantial barrage of excitatory input to spinal dorsal horn and then to supraspinal regions and contribute to behavioral hypersensitivity associated with cervical radiculopathy.

Several ionic currents have been implicated in the generation of spontaneous activity and hyperexcitability in DRG neurons in various pathological states, including A-type and delayed rectifier type K^+^ currents^45^, TTX resistant Na^+^ currents^46^, and Ca^2+^ currents^47^. In the present study, however, we demonstrated that CRP is associated with increased *I*_h_ density in IB4^−^ Aδ-fiber, but not in IB4^−^ or IB4^+^ C-fiber nociceptive DRG neurons. Blockade of *I*_h_ could eliminate the generation of spontaneous activity occurred in IB4^−^ Aδ neurons from CRP rats. This increased *I*_h_ is strongly supported by upregulation of HCN1 and HCN3 in the IB4^−^ small diameter DRG neurons. Given the fact that HCN1, HCN2, HCN3 channels activate with a V_1/2_ of approximately −70 mV, −95 mV, –77 mV to −95 mV, respectively^48^,^49^,^50^,^51^, increased HCN1 and HCN3 expression is consistent with the right-shift of V_1/2_ of activation of *I*_h_ in the CRP IB4^−^ Aδ neurons compared to control neurons. The difference of V_1/2_ in our study and previous reports might be due to different tissues and experimental parameters (e.g. pulse protocol, temperature) and the intracellular milieu (e.g. pH, concentration of modulatory factors), since previous studies have shown that the activation time constants and voltage-dependence of HCN channels are strongly influenced by the above parameters^48^. This intrinsic sensitivity may explain some of the variability of biophysical parameters that have been found for the same HCN channels by different laboratories. Consistent with our result, enhancement of *I*_h_ density has been found in medium-sized DRG neurons derived from compressed lumbar DRGs model^52^. In striking contrast, chronic inflammation induced an upregulation of *I*_h_ in C- but not Aδ-nociceptors, which is dependent on increased expression of HCN2 channels^30^. These results inferred us that enhancement of *I*_h_ density in IB4^−^ Aδ neurons contributes, at least in partial, to the spontaneous activity and hyperexcitability associated with CRP.

Aberrant mechanical hypersensitivity is a frequently-seen pain symptom associated with cervical radiculopathy. However, its underlying mechanism remains elusive. Numerous studies have reported that DRG neuron somata respond to mechanical stimulation^13^,^14^,^15^. Whether and how this neuronal mechanosensitivity is altered under pathological states are poorly understood. Another striking finding of the present study is that IB4^−^ Aδ-fiber DRG neurons exhibited marked plastic changes in mechanosensitivity, namely mechanical hypersensitivity in CRP rats. Furthermore, this neuronal mechanical hypersensitivity was followed by long-lasting afterdischarges. To the best of our knowledge, this is the first study to show activity-dependent upregulation of Aδ-type nociceptive DRG neuron somata to mechanical stimulation under pathological states. Such increased mechanical response together with afterdischarges as well as spontaneous firing could cause exaggerated inflow of painful signals to the CNS and thus resulting in behavioral mechanical hypersensitivity associated with CRP.

The possible involvement of mechanotransducers such as Piezo or TRPC channels in the development of mechanical hypersensitivity has been a topic of interest^53^,^54^. More recently, it has been reported that Epac1 potentiation of Piezo2-mediated mechanotransduction contributes to mechanical allodynia during neuropathic pain^15^. However, in our present study, we showed that increased *I*_h_ current mediated the mechanical hypersensitivity in IB4^−^ Aδ-fiber DRG neuron somata and further behavioral mechanical hypersensitivity observed in CRP. It should be pointed that the mechanism underlying the role of *I*_h_ in the neuronal mechanical hypersensitivity will be required for further investigation in the future study.

In summary, our results revealed a novel hypothesis that mechanical hypersensitivity, in a model of cervical radiculopathy in the rat, is mediated at least in part by exaggerated mechanical response in IB4^−^ Aδ-fiber neurons, which is dependent on increased *I*_h_ current. Therefore, targeting *I*_h_ current in IB4^−^ Aδ-fiber neurons might represent a new potential therapeutic candidate for the treatment of chronic pain, including cervical radiculopathy.

## Methods

### Animal and Surgery

Adult Sprague-Dawley rats weighing 100–150 g were subjected to CRP operation. All experimental protocols were approved by the Institutional Animal Use and Protection Committee, Fourth Military Medical University. All the testing was carried out in accordance with the approved guidelines. Briefly, the skin was incised on the left side along cervical vertebrae and the left paraspinal muscles separated from the mammillary process and the transverse process. The intervertebral foramen of vertebrae C_6–7_ and vertebrae C_7_T_1_ was clearly exposed. A fine, stainless L-shaped (at an angle of 60°) steel rod (3 mm in length, 0.63 mm in diameter) inserted into intervertebral foramen for steady compression of corresponding DRGs at a rostral direction at an angle of 90° to the dorsal middle line (Fig. 1a). After the rods were in place, the muscle and skin layers were sutured with administration of about 200 mg Penicillin antibiotic. Animals were mildly kept on a heating pad after operation to keep the body temperature till they became awake. The operated animals were housed in plastic boxes separately with food and water available ad libitum in the colony room.

### Behavioral tests

Behavioral testing was carried out in habituated rats by an observer blinded to the identity of the groups. Mechanical sensitivity was tested with manual application of Von Frey hairs to the plantar surface of forepaw. Each filament was applied 10 times and the paw withdrawal response frequency (the percentage of positive responses to the stimulus) was recorded. The force of a particular filament required to elicit 50% frequency of paw withdrawal was expressed as the mechanical threshold. Thermal sensitivity was tested by application of infrared heat to the plantar surface of forepaw and the response latency was measured from an automated device readout, as described previously^55^,^56^. To test spontaneous pain behavior, the rat was placed on the surface of a 2 mm thick glass covered by a transparent Plexiglas box. The spontaneous pain was determined by counting the number of seconds the rat spent in lifting, licking and biting the injured forepaw during 1 min interval every 20 min. The mean duration of the spontaneous pain in an hour was recorded.

### Immunofluorescence labeling

Rats were perfused with 4% paraformaldehyde (PFA) and DRG, spinal cord were extracted and postfixed overnight in 4% PFA. Immunofluorescence staining was performed on cryosections (16 μm) using standard reagents and protocols. Briefly, the sections were incubated with a solution containing 0.3% Triton X-100 and 1% bovine serum albumin (BSA) for 30 min at room temperature. The sections then were incubated with rabbit polyclonal anti-c-fos antibody (1:10000, Cell Signaling Technology), rabbit anti-phospho-p44/42 Map Kinase (Thr202/Tyr204) antibody (1:200, Cell Signaling Technology) for 24 h. After three rinses, the sections were further incubated with the secondary antibodies Alexa Fluor 488 (goat anti-rabbit IgG) (Invitrogen) for 2 h at room temperature. All images were captured with an Olympus confocal microscope and processed with Adobe Photoshop software.

DRG and spinal cord sections with c-Fos and phospho-ERK (pERK1/2) positive components from each animal were selected randomly from C7–8 segments. For DRG section, the percentage of Fos- and pERK1/2-positive cells in the number of small cells was quantified per section. For spinal cord section, the number of Fos- and pERK1/2-positive cells in the superficial (lamina I and II) and deep (lamina IV-VI) layers of spinal dorsal horn per section was counted, respectively. Data was averaged from at least eight sections/rat, with totally 3 rats per group.

### Intact DRG preparations and whole cell patch clamp recording

C7 and C8 DRGs with attached nerve were carefully removed at postoperative 3–5d from CRP rats or control rats. After removing the connective tissue, the ganglia were digested with a mixture of 0.4 mg/mL trypsin (Sigma) and 1.0 mg/ml type-A collagenase (Sigma) for 45 min at 37 °C. The intact ganglia were then incubated in ACSF oxygenated with 95% O_2_ and 5% CO_2_ at 28 °C for at least 1 h before transferring them to the recording chamber^42^. DRG neurons were visualized with a 40X water-immersion objective using a microscope (BX51WI; Olympus, Tokyo, Japan) equipped with infrared differential interference contrast optics. Whole-cell current and voltage recordings were acquired with an Axon700B amplifier (Molecular Devices Corporation, Sunnyvale, CA, USA). Patch pipettes (4–7 MΩ) were pulled from borosilicate glass capillaries on P-97 puller (Sutter Instruments, USA). The series resistance was 10–20 MΩ. Neurons were selected for further study if they had a resting membrane potential negative than −50 mV and if they exhibited overshooting action potentials.

The recorded small DRG neurons (≤25 μm) were classified by both the conduction velocity (CV) and IB4 binding. Neurons with CV less than 0.8 m/s were classified as C-type nociceptive neurons, CV at 1.5–6.5 m/s as Aδ-fiber nociceptive neurons, and CV more than >6.5 m/s as Aα/β (>6.5 m/s) as previously described^57^. In brief, the conduction velocity of each neuron was determined by dividing the length of dorsal root attached by the latency between stimulus artifact and the onset of the evoked somatic action potential (AP). The AP threshold was determined by differentiating the AP waveform and setting a rising rate of 10 mV/ms as the AP inflection point. The AP amplitude was measured from the equipotential point of the threshold to the spike peak. For live identification of IB4 binding, neurons were incubated with a vital marker, Alexa 488 conjugated IB4 (10 μg/ml; Molecular probes) as described by Luo *et al*.^16^ and washed in bathing solution before recording.

The ACSF contained (in mM): 124 NaCl, 2.5 KCl, 1.2 NaH_2_PO_4_, 1.0 MgCl_2_, 2.0 CaCl_2_, 25 NaHCO_3_, and 10 Glucose. The pipette solution contained (in mM): 140 KCl, 2 MgCl_2_, 10 Hepes, 2 Mg-ATP, pH 7.4. Osmolarity was adjusted to 290–300 mOsm. All chemicals were obtained from Sigma, St. Louis, MO, USA. Data was acquired with a digidata 1322A acquisition system (molecular devices) using pCLAMP 9.0 software. Signals were low-pass filtered at 5 kHz, sampled at 10 kHz and analyzed offline.

### Mechanically activated property recording

Focal mechanical stimulus was applied to DRG somata using a heat-polished glass pipette. The pipette was controlled by a manual manipulator and positioned at the opposite of the recording pipette. For stimuli, the pipette moved towards the membrane of the somata at low speed shift lasting for 1 s, then removed. The distance the pipette moved was 1/6 diameter of the recorded small neuron (about 5–7 μm) with an observable membrane deflection. The recorded cell would be rest for 5–10 min for the interval to reinitiate stimuli. While stimulating, (1) spikes with overshot above 0 mV; (2) discharge frequency increased 200% or more would be regarded as mechanically evoked events. And the event ended if (1) the spikes stopped; (2) the discharge frequency dropped to 10% of that in the event or to the beginning level of spontaneous activity.

### *I*_h_ Current Analysis

For recording of *I*_h_, ACSF and normal intrapipette solution mentioned above were used. To activate *I*_h_, hyperpolarizing potentials of −50 to −120 mV were delivered in increments of 10 mV from a holding potential of −60 mV for duration of 3.5 s. The current was obtained by the steady state current minus current at the beginning of the step. Tail current analysis was taken to analyze the steady state inactivation of *I*_h_.

### Intrathecal administration

The rats were anaesthetized by 1% pentobarbital sodium. A midline incision was made along T8 to L2 and the muscle attached to spinous process removed. With the tip of the sharp scissor, a 1-mm hole on the right vertebra was made until dura and clean CSF was exposed. An intrathecal catheter (polyethylene-10 tubing) was inserted from T8 and passed rostrally into the subarachnoid space until it reached C7/C8. After a flush with 10 μl artificial CSF, the exterior end of catheter was sealed by heat. The rats were allowed to recover for 3 days. Any rat showing motor deficits would be excluded. Those without motor deficits underwent CRP operation further. At 3 d after CRP operation, ZD7288 was intrathecally applied in a volume of 5 μl followed by a 5 μl artificial CSF flush. Penicillin antibiotics were used to prevent infection at the end of both intrathecal catheterization and CRP surgery. Animals were mildly kept on a heating pad after operation to keep the body temperature till they became awake. The operated animals were housed in plastic boxes separately with food and water available ad libitum in the colony room.

### Statistical Analysis

All data are expressed as mean ± S.E.M. Student’s t-test or an analysis of variance (ANOVA) was carried out, followed by either a post hoc Fisher’s test or Dunnett’s test. *P* < 0.05 was considered significant.

## Additional Information

**How to cite this article**: Liu, D.-L. *et al*. Upregulation of *I*_h_ expressed in IB4-negative Aδ nociceptive DRG neurons contributes to mechanical hypersensitivity associated with cervical radiculopathic pain. *Sci. Rep*. **5**, 16713; doi: 10.1038/srep16713 (2015).
